# Utility values for glaucoma patients in Korea

**DOI:** 10.1371/journal.pone.0197581

**Published:** 2018-05-30

**Authors:** Seulggie Choi, Jin A. Choi, Jin Woo Kwon, Sang Min Park, Donghyun Jee

**Affiliations:** 1 Seoul National University Graduate School, Department of Biomedical Sciences, Seoul, Republic of Korea; 2 St. Vincent’s Hospital, College of Medicine, Division of Vitreous and Retina, Department of Ophthalmology, Catholic University of Korea, Suwon, Republic of Korea; 3 Seoul National University Hospital, Department of Family Medicine, Seoul, Republic of Korea; Universita degli Studi di Firenze, ITALY

## Abstract

**Objectives:**

Although determining the quality of life among glaucoma patients has important clinical and public health implications, the utility value of glaucoma patients has not yet been determined in Korea.

**Methods:**

The Korean National Health and Nutrition Examination Survey database was used to identify 833 glaucoma patients based on ophthalmologic examinations. The adjusted mean utility values, calculated by EuroQol-5D (EQ-5D-3L), of glaucoma patients according to patient demographics and measures of glaucoma severity were determined using multivariate linear regression analysis.

**Results:**

The mean utility value of glaucoma patients was 0.8968. Patients aged 70 years or more had significantly lower utility value (0.86, *p* value 0.005) compared to those aged less than 50 years (0.96). Patients within the lowest quartile of income had a utility value of 0.87, compared to a utility value of 0.96 for those within the highest quartile (*p* value 0.001). Patients who were not married had lower utility value (0.87) compared to married patients (0.93). Patients within the lowest quartile of worse eye frequency doubling technology (FDT) score had lower utility value (0.88) compared to those within the highest quartile (0.94). Finally, bilateral vision loss patients had significantly lower utility value (0.83, *p* value 0.013) compared to patients without vision loss (0.92).

**Conclusion:**

The present study assessed utility values of Korean glaucoma patients. The quality of life determined by EQ-5D-3L in Korean glaucoma patients was higher compared to those in other countries. Patient demographics as well as measures of disease severity were important factors in determining the quality of life within glaucoma patients.

## Introduction

Glaucoma is the second leading cause of blindness in the world, affecting more than 70 million people [1]. Particularly, the number of glaucoma patients is expected to reach 37 million by 2020 in Asia alone, constituting 47% of the world’s population of glaucoma patients [1]. Due to the increased life expectancy, the prevalence of glaucoma has increased in Korea, with recent studies reporting prevalence rates of 3.5% [2] and 4.5% [3] for POAG and PACG, respectively. Furthermore, the prevalence of glaucoma in Korea is expected to continue to increase, with a recent study reporting a 54% increase in glaucoma prevalence from 2008 to 2013 [4].

One of the primary goals of managing glaucoma is maintaining the quality of life of the patients at a socially acceptable cost. Determining the quality of life for patients with a disease of rising prevalence has important clinical and public health implications [5]. Utility value, which determines a patient’s perception of the quality of life from a scale of 0 to 1, quantifies the strength of one’s preference for a health state. Furthermore, utility values can be used to compare the quality of life among different groups and are thus useful in determining the impact a disease has on the health status of patients.

While previous studies have determined the utility values of glaucoma patients in a number of different countries [6], there are limited studies investigating the quality of life of glaucoma patients in Korea. Therefore, we used a nationwide representative survey to determine utility values of glaucoma patients as a part of the economic evaluation of glaucoma management in Korea.

## Methods

### Study population

We used the Korean National Health and Nutrition Examination Survey (KNHANES) database conducted from 2008 to 2012 for this study. KNHANES is a nationwide population-based cross-sectional survey consisting of health records from health interviews and examinations [7]. From 2008 to 2012, the Korean Ophthalmologic Society participated in KNHANES, thereby including ophthalmologic interviews and examinations conducted by trained ophthalmologists within the survey [8].

For the evaluation of glaucoma, slit-lamp examinations and intraocular pressure (IOP) measurement using a Goldmann applantation tonometer were conducted. A digital non-mydriatic fundus camera (TRC-NW6S, Topcon) and a digital camera (Nikon D-80, Nikon Inc., Tokyo, Japan) were used to capture digital fundus images of all participants under physiological mydriasis. If the participants had an IOP of greater than or equal to 22 mmHg or a glaucomatus optic disc, visual field testing using the frequency doubling technology (FDT) Humphrey Matrix (Carl Zeiss Meditec Inc., Dublin, CA, USA) was conducted. A glaucomatous optic disc was defined as having: (a) a vertical or horizontal cup-to-disc ratio of 0.5 or greater, (b) a retinal nerve fiber layer defect, (c) an optic disc hemorrhage, or (d) violated the ISNT (inferior, superior, nasal, and temporal) rule. Finally, visual acuity was measured at 4 meters with an international standard vision chart based on the LogMAR Scale (Jin’s Vision Chart, Seoul, Korea).

Glaucoma was defined according to the International Society for Geographical and Epidemiological Ophthalmology diagnostic criteria [9, 10]. Specifically, glaucoma was defined as the presence of optic nerve damage (vertical or horizontal cup-to-disc ratio of 0.6 or greater, disc hemorrhage, or retinal nerve fiber layer defect) and an abnormal FDT testing result. Among glaucoma patients, primary open angle glaucoma (POAG) patients were defined as those with a peripheral anterior chamber depth of greater than 1/4^th^ of the corneal thickness, while primary angle-closure glaucoma (PACG) patients were defined as those with a peripheral anterior chamber depth of 1/4^th^ of the corneal thickness or less. FDT score was calculated by adding the number of abnormal locations from FDT testing, after which the scores were divided into quartiles. Finally, vision loss was defined when visual acuity was less than 0.1 [11].

Among the 27,088 participants aged 19 years or more with available data on slit-lamp examinations and fundus images, 26,230 participants who did not meet the criteria for having glaucoma were excluded, resulting in 858 glaucoma patients. Among them, 12 participants without values on education status and 13 participants without values on household income were excluded, ultimately resulting in a study population of 833 glaucoma patients.

### Measurement of utility value

Developed by the EuroQol Group, EQ-5D-3L is a generic preference-based measure consisting of five questions that reflect the current health status of the patient [12, 13]. The questions are composed of five dimensions: mobility, self-care, usual activities, pain/discomfort, and anxiety/depression. Each question has three levels that indicate (a) no problem, (b) some problems, or (c) severe problems. EQ-5D-3L have been used widely as a tool of assessing the health status. The Korean EQ-5D-3L questionnaire was developed following the EuroQol group procedure by the Korean Centers for Disease Control and Prevention [14]. The Korean EQ-5D-3L utility score ranges from -0.171 (worst health status) to 1.000 (best health status)

### Demographics and measures of glaucoma severity

Among patient demographics, age (years, less than 50, 50–59, 60–69, and 70 or more), sex (men and women), education (elementary school or lower, middle school, high school, technical college, and college or higher), employment status (yes and no), household income (1^st^, 2^nd^, 3^rd^, and 4^th^ quartiles), and marital status (yes and no) were determined by a questionnaire. Glaucoma subtype (POAG and PACG), better eye cup-to-disc ratio (<0.6 and ≥0.6), better and worse eye FDT score (1^st^, 2^nd^, 3^rd^, and 4^th^ quartiles), and vision loss (no vision loss, unilateral vision loss, and bilateral vision loss) were determined.

### Statistical analysis

The proportions of the patient demographics and measures of glaucoma severity were calculated. For each patient demographic and measure of glaucoma severity, the adjusted means and 95% confidence intervals (CI) of EQ-5D-3L utility values were calculated. Adjusted mean values were calculated by linear regression analysis. In Model 1, covariates age and sex were adjusted. In Model 2, education, employment status, household income, martial status, glaucoma subtype, better eye cup-to-disc ratio, and vision loss were additionally adjusted. Statistical significance was assumed at a *p* value of less than 0.05 in a two-sided manner. All statistical analyses conducted in this study were done with STATA 13.0 (StataCorp LP, College Station, TX, USA).

### Ethical considerations

All participants of KNHANES from 2008 to 2012 provided informed consent before the survey. No approval from the Institutional Review Board was needed as KNHANES is publicly available from the Korea Centers for Disease Control and Prevention.

## Results

Table 1 depicts the descriptive characteristics of the study population. The mean utility value (standard deviation) was 0.8968 (0.1597). Among 833 glaucoma patients, the mean age was 61.3 years. There were similar numbers of men (416 patients, 49.1%) and women (417 patients, 50.1%). For education, the greatest proportion of the highest level of education was elementary school or lower (35.2%), while 17.3% of the patients graduated from college or higher. Most patients were POAG patients (809 patients, 97.1%), while only 24 patients (2.9) were PACG patients. Finally, the proportions of patients with no vision loss, unilateral vision loss, and bilateral vision loss were 95.3%, 4.3%, and 0.4%, respectively.

**Table 1:** Descriptive characteristics of the study population.

Descriptive characteristics (n = 833)	
EQ-5D-3L score, mean (SD)	0.8968 (0.1597)
Age, years, mean (SD)	61.3 (14.0)
Age, years, N (%)	
Less than 50	166 (20.5)
50–59	159 (19.6)
60–69	237 (29.2)
70 or more	249 (30.7)
Sex, N (%)	
Men	416 (49.9)
Women	417 (50.1)
Education, N (%)	
Elementary school or lower	293 (35.2)
Middle school	139 (16.7)
High school	176 (21.1)
Technical college	81 (9.7)
College or higher	144 (17.3)
Employment status, N (%)	
Yes	419 (50.3)
No	414 (49.7)
Household income, N (%)	
1^st^ quartile (lowest)	343 (41.2)
2^nd^ quartile	189 (22.7)
3^rd^ quartile	146 (17.5)
4^th^ quartile (highest)	155 (18.6)
Marital status, N (%)	
Yes	793 (95.2)
No	40 (4.8)
Glaucoma subtype, N (%)	
POAG	809 (97.1)
PACG	24 (2.9)
Better eye cup-to-disc ratio, N (%)	
<0.6	392 (47.1)
≥0.6	441 (52.9)
Better eye FDT score, N (%)	
1^st^ quartile (best)	247 (29.7)
2^nd^ quartile	208 (25.0)
3^rd^ quartile	188 (22.6)
4^th^ quartile (worst)	190 (22.8)
Worse eye FDT score, N (%)	
1^st^ quartile (best)	317 (38.1)
2^nd^ quartile	150 (18.0)
3^rd^ quartile	180 (21.6)
4^th^ quartile (worst)	186 (22.3)
Vision loss, N (%)	
No vision loss	794 (95.3)
Unilateral vision loss	36 (4.3)
Bilateral vision loss	3 (0.4)

Acronyms: EQ-5D-3L, three level version of EuroQol-5D; SD, standard deviation; POAG, primary open angle glaucoma; PACG, primary angle-closure glaucoma; FDT, frequency doubling technology

The adjusted mean utility values according to sociodemographic characteristics are shown in Table 2. When the utility values were determined according to age, the adjusted mean EQ-5D-3L score for the youngest group (less than 50 years) was 0.96 (95% CI 0.94–0.99), while the mean EQ-5D-3L score for the oldest group (70 years or more) was 0.86 (95% CI 0.80–0.92). There was a statistically significant trend towards decreased utility values with increasing age (*p* for trend 0.005). The adjusted mean utility value for patients within the lowest quartile of household income was 0.87 (95% CI 0.83–0.90) while patients within the highest quartile had an adjusted utility value of 0.96 (95% CI 0.93–0.99). Increasing quartiles of household income was associated with improved utility values (*p* for trend <0.001). Finally, patients who were not married had significantly lower adjusted mean utility values (0.87, *p* value 0.014) compared to married patients (0.93).

**Table 2:** Utility values according to age, sex, education, employment status, household income, and martial status.

	EQ-5D-3L Adjusted mean (95% CI)		
Category	Model 1	Model 2	*p* value
Age			
Less than 50 years	0.97 (0.95–0.99)	0.96 (0.94–0.99)	reference
50–59 years	0.95 (0.92–0.98)	0.93 (0.91–0.96)	0.077
60–69 years	0.88 (0.83–0.92)	0.90 (0.86–0.94)	0.008
70 years or more	0.83 (0.77–0.89)	0.86 (0.80–0.92)	0.009
*p* for trend			0.005
Sex			
Men	0.93 (0.91–0.96)	0.93 (0.90–0.95)	reference
Women	0.92 (0.89–0.94)	0.93 (0.90–0.95)	0.983
Education			
Elementary school or lower	0.86 (0.77–0.95)	0.92 (0.83–1.01)	reference
Middle school	0.89 (0.85–0.94)	0.91 (0.87–0.95)	0.837
High school	0.92 (0.86–0.98)	0.92 (0.87–0.97)	0.973
Technical college	0.94 (0.92–0.96)	0.92 (0.90–0.95)	0.944
College or higher	0.95 (0.93–0.98)	0.94 (0.92–0.97)	0.664
*p* for trend			0.283
Employment status			
Yes	0.92 (0.90–0.94)	0.92 (0.90–0.94)	reference
No	0.93 (0.90–0.96)	0.93 (0.90–0.96)	0.819
Household income			
1^st^ quartile (lowest)	0.86 (0.82–0.91)	0.87 (0.83–0.90)	reference
2^nd^ quartile	0.94 (0.91–0.97)	0.94 (0.91–0.97)	0.005
3^rd^ quartile	0.96 (0.94–0.99)	0.97 (0.94–0.99)	<0.001
4^th^ quartile (highest)	0.97 (0.94–0.99)	0.96 (0.93–0.99)	0.001
*p* for trend			<0.001
Marital status			
Yes	0.93 (0.92–0.95)	0.93 (0.92–0.95)	reference
No	0.87 (0.82–0.93)	0.87 (0.83–0.92)	0.014

Model 1: adjusted mean values calculated using linear regression analysis after adjustments for age and sex

Model 2: additionally adjusted for education, employment status, household income, martial status, glaucoma subtype, better eye cup-to-disc ratio, FDT score, and vision loss

Acronyms: EQ-5D-3L, three level version of EuroQol-5D; CI, confidence interval

Table 3 demonstrates the adjusted mean utility values according to glaucoma subtype, better eye cup-to-disc ratio, FDT score, and vision loss. Compared to POAG patients, PACG patients had significantly higher adjusted mean utility values (*p* value 0.012). Furthermore, while the utility value for patients within the first quartile of FDT score in the worse eye was 0.94 (95% CI 0.91–0.96), the utility value for those within the fourth quartile was 0.88 (95% CI 0.84–0.92), with a *p* value of 0.024. Finally, patients with no vision loss had an adjusted mean utility value of 0.92 (95% CI 0.91–0.94), while patients with bilateral vision loss had a mean utility value of 0.83 (95% CI 0.76–0.90). Patients with bilateral vision loss had significantly lower utility values compared to patients without vision loss (*p* value 0.013).

**Table 3:** Utility values according to glaucoma subtype, better eye cup-to-disc ratio, FDT score, and vision loss.

	EQ-5D-3L Adjusted mean (95% CI)		
Category	Model 1	Model 2	*p* value
Glaucoma subtype			
POAG	0.92 (0.91–0.94)	0.92 (0.91–0.94)	reference
PACG	0.98 (0.94–1.01)	1.01 (0.94–1.07)	0.012
Better eye cup-to-disc ratio			
<0.6	0.93 (0.90–0.96)	0.93 (0.90–0.96)	reference
≥0.6	0.92 (0.90–0.95)	0.92 (0.91–0.94)	0.736
Better eye FDT score			
1^st^ quartile (best)	0.93 (0.90–0.97)	0.94 (0.91–0.96)	reference
2^nd^ quartile	0.93 (0.91–0.96)	0.92 (0.90–0.95)	0.403
3^rd^ quartile	0.92 (0.88–0.93)	0.92 (0.89–0.96)	0.483
4^th^ quartile (worst)	0.92 (0.87–0.96)	0.92 (0.89–0.96)	0.521
*p* for trend			0.521
Worse eye FDT score			
1^st^ quartile (best)	0.94 (0.92–0.97)	0.94 (0.91–0.96)	reference
2^nd^ quartile	0.94 (0.91–0.97)	0.94 (0.91–0.97)	0.951
3^rd^ quartile	0.93 (0.89–0.97)	0.94 (0.90–0.97)	0.942
4^th^ quartile (worst)	0.87 (0.82–0.92)	0.88 (0.84–0.92)	0.024
*p* for trend			0.067
Vision loss			
No vision loss	0.93 (0.91–0.94)	0.92 (0.91–0.94)	reference
Unilateral vision loss	0.98 (0.87–1.09)	1.02 (0.92–1.13)	0.062
Bilateral vision loss	0.70 (0.67–0.73)	0.83 (0.76–0.90)	0.013
*p* for trend			0.320

Model 1: adjusted mean values calculated using linear regression analysis after adjustments for age and sex

Model 2: additionally adjusted for education, employment status, household income, martial status, glaucoma subtype, better eye cup-to-disc ratio, FDT score, and vision loss

Acronyms: EQ-5D-3L, three level version of EuroQol-5D; CI, confidence interval; POAG, primary open angle glaucoma; PACG, primary angle-closure glaucoma; FDT, frequency doubling technology

## Discussion

We have shown that the mean EQ-5D-3L score of 833 glaucoma patients in Korea was 0.8968 (SD 0.1597). Glaucoma patients who are older, have low income status, and are not married had lower utility values. Finally, glaucoma patients with PACG, more advanced degrees of glaucoma severity, and with vision loss had lower utility values.

Several previous studies have used EQ-5D to determine utility values for glaucoma patients [15–18]. Aspinall and colleagues, who investigated the quality of life of glaucoma patients, determined that the mean EQ-5D score for 72 glaucoma patients in the United Kingdom was 0.76 (SD 0.19) [15]. Another study comparing the sensitivity of EQ-5D, Short Form-6D, and Time Trade Off utility values among POAG patients showed that the mean EQ-5D score for 131 POAG patients in the United Kingdom was 0.8 (SD 0.2) [16]. Similarly, two separate studies evaluating utility values among glaucoma patients showed mean EQ-5D scores for glaucoma patients in Sweden and Europe were 0.80 (SD 0.23) and 0.65 (SD 0.28), respectively [17, 18].

Interestingly, the utility values for glaucoma patients in the United Kingdom, Sweden, and Europe were all lower compared to that among Korean glaucoma patients determined in our study. One possible contributing factor is the active promotion of awareness for glaucoma by a number of ophthalmologic societies in Korea. This could increase the awareness of glaucoma among the general population and thus make it more likely for early diagnosis. Earlier diagnosis of glaucoma may lead to easier management of the disease and thus yield less symptoms and complications. Furthermore, increased awareness may prompt glaucoma patients to adhere to medications and management regimens, thereby yielding more favorable outcomes. However, the exact reasons for the higher utility values among Korean glaucoma patients compared to those in other countries cannot be determined from our results and merit further investigation.

A number of previous studies have determined utility values for glaucoma patients according to patient demographics such as age, income, sex, and education [19–21]. In two separate studies, there was no significant difference in utility values according to age among glaucoma patients (*p* values 0.69 and 0.46, respectively) [20, 21]. In contrast, we found that older glaucoma patients had lower utility scores compared to younger patients (*p* for trend 0.005). While the reasons for the differing results of utility values according to age are unknown, it is reasonable to assume that older glaucoma patients may have been diagnosed with glaucoma for longer durations, likely resulting in more advanced stages of the disease. Greater degrees of glaucoma progression or severity among older patients may result in more symptoms and complications, contributing to the lower utility values compared to young patients. The reasons as to why utility values did not differ according to age among glaucoma patients in previous studies are unclear and merit further evaluation.

In contrast to the results from our study, a previous study investigating the utility values among glaucoma patients revealed that there was no significant difference in utility values according to income [21]. Although the reasons for the discrepancy between studies in the association of income and utility value among glaucoma patients are unclear, it is reasonable to assume that patients with higher income may have more regular access to high quality care, which could result in better management of glaucoma and improved quality of life. Among previous studies that examined utility values for glaucoma patients according to sex, one found no difference in utility values [19], another showed that men had lower utility values [20], while another revealed that women had lower utility values [21]. In our study, we found that there was no significant difference in utility values for men and women.

We could not find any significant difference in utility values according to education, which is in contrast to a previous study also showing lower utility values for those with lower levels of education among 213 Chinese glaucoma patients [20]. Although patients with higher education may be expected to have higher utility values as such patients may be more self-aware of the early signs of glaucoma and thus may be more likely to be diagnosed early, our results suggest that other factors highly correlated with education, such as household income, may act more strongly on utility values among glaucoma patients. Aside from age and income, we have added to previous studies by showing that utility values according to martial status are also different.

Similar to the results from our study, a previous study has shown that PACG patients had better utility values compared to POAG patients [22]. This may be due to the fact that POAG and PACG are different in how the symptoms are presented. While POAG patients tend to have no symptoms until the disease has progressed, PACG patients tend to have immediate symptoms such as eye pain and blurred vision. Due to the acute nature in which PACG symptoms are presented, PACG patients may seek early medical care, which could result in improvement of symptoms [23, 24]. In contrast, POAG patients suffer from the relatively delayed presentation of symptoms and thus may be associated with lower utility values compared to PACG patients.

Previous studies have shown that utility values differ according to glaucoma severity in terms of visual acuity, mean deviation index, and pattern standard deviation [19, 20]. Similarly, we have shown that utility values according to FDT score and vision loss are different among glaucoma patients. As FDT score may be considered as a measure of the degree of visual field seeing capacity, glaucoma patients with advanced cases of glaucoma may have lower utility values. Similarly, as visual acuity is directly related to the ability to conduct everyday activities, patients with bilateral vision loss could result in decreased quality of life.

There are several limitations to consider when interpreting the results of this study. First, the cross-sectional nature of this data makes it difficult to rule out the possibility of reverse causality, in which decreased utility values may be the cause of differences in patient demographics, rather than the other way around. Second, EQ-5D is known to have a ceiling effect problem in which as much as 65% of the general population report perfect EQ-5D scores [25]. Therefore, studies using other tools of measuring utility are needed to confirm the findings of this study. Third, visual field defect was determined by the FDT Humphrey Matrix, rather than the Zeiss-Humphrey field analyzer as suggested in guidelines [9]. Use of the FDT Humphrey Matrix may have led to an overestimation of glaucoma patients, leading to a subsequent greater utility value for glaucoma patients compared to that in previous studies. Furthermore, mean deviation could not be determined, which is an important indicator of glaucoma progression and future studies using the Zeiss-Humphrey field analyzer are needed to further validate the findings of this study. Finally, other serious comorbidities that may affect the utility values of glaucoma patients were not considered. However, a previous study has shown that systemic comorbidities does not have an impact on the quality of life of patients with ophthalmologic diseases [26].

Despite these limitations, our study was based on a nationally representative data with a study population of 833 glaucoma patients, which is larger than most previous studies. Furthermore, we took into account a greater number of demographics, such as employment status, household income, and marital status, which few previous studies have investigated upon. Finally, despite its ceiling effect problem, EQ-5D is nevertheless a widely used tool for measuring utility values and the results of this study could later be used as a primary source of QALY estimation for cost-effective analyses.

## Conclusions

The utility value for glaucoma patients in Korea was higher compared to glaucoma patients in other countries. Glaucoma patients who are older, have low income status, and are not married had lower utility values. Furthermore, glaucoma patients who were diagnosed with PACG, with a greater degree of disease severity, and with bilateral vision loss had lower quality of life.
